# How Benzodiazepines Affect Cognition in Patients with Schizophrenia

**DOI:** 10.1192/j.eurpsy.2026.12146

**Published:** 2026-07-31

**Authors:** D. Njah, M. Lagha, A. Zyed, W. Homri

**Affiliations:** Psychiatry c, Razi psychiatric hospital, Manouba, Tunisia

## Abstract

**Introduction:**

Schizophrenia is frequently associated with marked cognitive impairment, affecting social, occupational, and daily functioning. Benzodiazepines are commonly prescribed in this population to manage symptoms such as anxiety, agitation, or insomnia. However, accumulating evidence indicates that benzodiazepine use may worsen cognitive dysfunction, potentially hindering recovery and functional outcomes. Examining the relationship between benzodiazepines and cognition is therefore crucial for optimizing treatment strategies in patients with schizophrenia.

**Objectives:**

Our objective was to assess the impact of benzodiazepine use on cognitive function in patients with schizophrenia and schizoaffective disorder (SAD).

**Methods:**

This was a cross-sectional study including patients with schizophrenia and SAD, aged between 18 and 65 years, that took place over a 2-month period, from February 1, 2025 to April 15, 2025 who were followed up at RAZI psychiatric Hospital in Tunisia. Cognitive function was assessed using the Arabic version of the Montreal Cognitive Assessment (MoCA) scale. Data on benzodiazepine use were collected from patients’ medical records.

**Results:**

We included 56 patients with schizophrenia and 14 patients with SAD. The mean age was 42 years (SD, 11). Sixty seven percent of our population were male(n=47) and almost 76% (n=53) were single. Among the patients, 53%(n=37) had been using benzodiazepines for at least one year, including 49% (n = 18) on lorazepam, 11% (n = 4) on diazepam, and 40% (n = 15) on other agents such as clonazepam or bromazepam. The median MoCA score was 20 [6-9]. Cognitive dysfunction was found in 74,3% of our population(n=52). A significant association was found between benzodiazepine use and cognitive dysfunction (p = 0.002), particularly with lorazepam (p = 0.013). Patients with lower MoCA scores were more likely to be taking benzodiazepines, and lorazepam use alone explained 20.2% of the variance in MoCA scores (R² = 0.202).

**Conclusions:**

These findings indicate that long term benzodiazepine use, particularly those with a long half-life such as lorazepam, is associated with greater cognitive impairment in patients with schizophrenia and SAD. Careful consideration of benzodiazepine prescriptions is therefore warranted to minimize potential negative effects on cognitive function in this population, emphasizing the need to restrict use to the minimal necessary duration.

**Disclosure of Interest:**

None Declared

